# Role of Peroxynitrite in the Pathogenesis of Parkinson’s Disease and Its Fluorescence Imaging-Based Detection

**DOI:** 10.3390/bios14100506

**Published:** 2024-10-17

**Authors:** Jiye Lv, Feiyu Chen, Changchan Zhang, Yubing Kang, Yan Yang, Chengwu Zhang

**Affiliations:** 1 School of Basic Medical Sciences, Shanxi Medical University, 56 Xinjiannan Road, Taiyuan 030001, China; 2 School of Chinese Medicine, Tianjin University of Traditional Medicine, 10 Poyanghu Road, Tianjin 301617, China

**Keywords:** Parkinson’s disease, peroxynitrite, pathogenesis, probe, fluorescence imaging

## Abstract

Parkinson’s disease (PD) is the second most common neurodegenerative disorder, affecting the lives of millions of people worldwide. Although the mechanism underlying PD pathogenesis is largely undefined, increasing evidence indicates that oxidative and nitrosative stresses play a crucial role in PD occurrence and development. Among them, the role of oxidative stress has been widely acknowledged, but there is relatively less attention given to nitrosative stress, which is mainly derived from peroxynitrite. In the present review, after briefly introducing the background of PD, we discuss the physiopathological function of peroxynitrite and especially highlight how overloaded peroxynitrite is involved in PD pathogenesis. Then, we summarize the currently reported fluorescence imaging-based peroxynitrite detection probes. Moreover, we specifically emphasize the probes that have been applied in PD research. Finally, we propose perspectives on how to develop a more applicable peroxynitrite probe and leverage it for PD theranostics. Conclusively, the present review broadens the knowledge on the pathological role of peroxynitrite in the context of PD and sheds light on how to develop and utilize fluorescence imaging-based strategies for peroxynitrite detection.

## 1. Introduction

Parkinson’s disease (PD) is the second most common neurodegenerative disorder, affecting the lives of millions of people worldwide, and the mechanism underlying PD pathogenesis is largely undefined. Increasing evidence indicates that oxidative and nitrosative stresses are crucial mediators of PD occurrence and development. Although the role of oxidative stress has been widely documented, there is relatively less attention given to nitrosative stress, which is mainly derived from peroxynitrite. In the present review, we first introduce the background of PD and the physiopathological function of peroxynitrite. We especially highlight the involvement of overloaded peroxynitrite in PD pathogenesis. Then, we summarize the currently reported fluorescence imaging-based probes for peroxynitrite detection. After that, we specifically review the probes that have been applied in PD research. Finally, we propose perspectives on how to develop a more applicable peroxynitrite probe and leverage it for PD theranostics. Hopefully, the present review can shed light on how to develop and utilize fluorescence imaging-based strategies for peroxynitrite detection in the context of PD.

## 2. Parkinson’s Disease

### 2.1. Epidemiology of PD

PD is the second most common neurodegenerative disease, characterized by the progressive loss of dopaminergic (DA) neurons in the substantia nigra compacta and the presence of Lewy bodies, mainly composed of aggregated alpha synuclein [1]. PD has become the fastest growing neurological disease in terms of incidence, disability, and mortality [2]. From 1990 to 2016, the incidence of PD increased by 21.7% [3]. It has been estimated that by 2040, the global number of PD patients will reach over 13 million [2,4]. The prevalence of PD varies significantly among populations in different regions, with the highest prevalence in high-income countries such as those in North America and Europe, and the lowest in low-income countries such as those in sub-Saharan Africa. Meanwhile, China has shown the largest increase in prevalence, which is five times higher than the global average [5]. The increasing aging population and deterioration of the environment brought on by industrialization could account for that dilemma [6,7].

### 2.2. Manifestation, Etiology, and Pathogenesis of PD

PD patients display both motor and non-motor manifestations. The typical motor symptoms of PD include static tremors, bradykinesia, stiffness of limbs and trunk, and postural instability [8]. There are also non-motor symptoms such as cognitive decline, fatigue, depression and anxiety, dementia, rapid eye movement sleep disorder, and taste and smell dysfunctions [9,10]. Notably, these non-motor symptoms may appear 15–20 years before the typical motor symptoms [11]. Currently, diagnosis of PD in clinics mainly depends on symptom checkup and bioimaging results (e.g., CT and PET). The latent features of PD and the high cost of bioimaging hinder its earlier diagnosis [12]. Despite the great efforts made by scientists and clinicians, there is no effective strategy to delay or prevent the progression of PD. Therefore, it is essential to determine the etiology and reveal the molecular mechanism of PD pathogenesis.

The etiology of PD is complicated and involves aging, environmental, and genetic factors, which exert impacts mostly in a synergistic manner. Aging is the most crucial causative factor of PD, with a ten times higher incidence in those aged above 65 years than in the young population [13]. Environmental factors such as heavy metals and pesticides, aggravated with industrialization, also enhance the onset of PD [14,15]. Genetic variation is thought to be a fundamental risk factor for PD, contributing to approximately 25% of overall PD cases. Mutations of single genes such as SNCA, Parkin, and LRRK2 are sufficient to cause familial PD, while most variations contribute to the onset of PD as risk factors [16]. Moreover, living habits such as alcoholism and smoking also exert an impact on the occurrence of PD [17,18,19]. Thus, the identification of causative factors could provide clues for the screening and diagnosis of PD.

**Figure 1:**
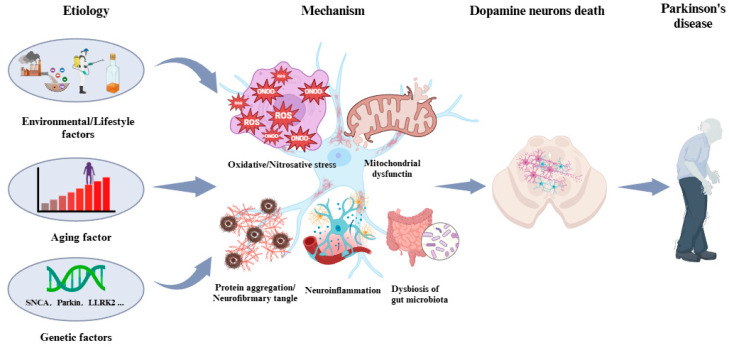
Etiology and pathogenesis of PD. The etiology of PD is attributed to aging, genetics, and environmental factors. The pathogenesis of PD is underlined by oxidative/nitrosative stress, mitochondrial dysfunction, aggregation and misfolding of proteins, neuroinflammation, and dysbiosis.

Mechanistically, the pathogenesis of PD is underlined by oxidative and nitrosative stress [20]; misfolding and aggregation of proteins, particularly alpha synuclein [21]; mitochondrial dysfunction; neuroinflammation [22]; and dysbiosis of gut microbiota [23], all of which work synergistically, leading to the death of DA neurons and the occurrence of PD [24] (Figure 1). As the key mediator of DA neuron death, oxidative stress has received much attention, and strategies to detect it and intervene are already widely reported [25]. However, nitrosative stress, caused mainly by peroxynitrite, has not received enough attention, though its role in PD onset and progression is significant [26].

## 3. Peroxynitrite and Its Involvement in PD 

### 3.1. Profile of Peroxynitrite

Peroxynitrite is a strong biological oxidant that plays an important role in homeostasis regulation and cellular signal transduction in both physiological and pathological conditions [27]. Peroxynitrite is formed by the diffusion-limited reaction of nitric oxide and superoxide anions [28]. In living systems, after performing its physiological roles, peroxynitrite can be cleared by antioxidants such as glutathione (GSH) and vitamins. However, when overloaded peroxynitrite is generated due to deleterious stimuli, it transforms the redox state of cells into a pro-oxidative state [29]. Excessive peroxynitrite disrupts intracellular redox homeostasis and causes oxidative damage to biomacromolecules such as DNA, proteins, and lipids [30]. DNA base nitrosylation by peroxynitrite leads to single strand breakage and initiates cell apoptosis. Protein nitrosylation, especially of tyrosine residues, alters the functions of proteins, which interrupts cellular signaling pathways and destroys mitochondrial function [31]. Ultimately, it triggers the onset of diseases such as neurodegenerative diseases, cancer, liver disease, rheumatism, etc. [32].

### 3.2. Involvement of Peroxynitrite in PD Pathogenesis

**Figure 2:**
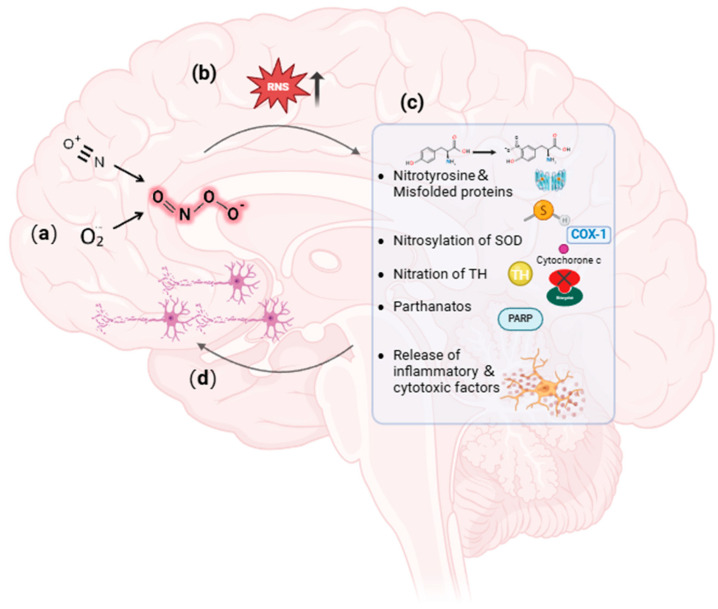
Role of peroxynitrite in the pathogenesis of PD. (**a**) Generation of peroxynitrite. (**b**) Accumulation of RNS. (**c**) Peroxynitrite leading to nitrosylation of proteins. (**d**) Peroxynitrite inducing DA neurons’ death.

As mentioned, excessive peroxynitrite would lead to the occurrence of various types of diseases. In the context of PD, mitochondrial dysfunction or the mutation of genes such as Parkin activates nitric oxide synthase (NOS), which triggers the generation of peroxynitrite [33]. The obtained peroxynitrite directly reacts with transition metals and catalyzes tyrosine residues to form nitrotyrosine, which alters the conformation of proteins and results in DA neurons’ death [34]. Peroxynitrite has been shown to induce the nitrosylation of superoxide dismutase (SOD), PARP, complex I, and cytochrome c, which propagates the production of reactive nitrogen species and further aggravates mitochondrial damage [35,36,37,38]. In addition, the nitration of tyrosine hydroxylase (TH), the rate-limiting enzyme of DA synthesis, not only depletes DA but also increases the production of active nitrogen, further leading to mitochondrial damage [39]. Simultaneously, oxidation of thiol groups and tyrosine disabilities of enzymes can also damage mitochondria [29,40,41]. Besides DA neurons, peroxynitrite also activates microglia in the substantia nigra and releases inflammatory and cytotoxic factors which contribute to PD pathogenesis [42,43] (Figure 2). 

Given the pivotal role of peroxynitrite in PD pathogenesis, it is of great significance to accurately detect peroxynitrite in vitro and in vivo so as to facilitate PD investigation and diagnosis. However, peroxynitrite is an inherently unstable isomer of nitrate with a half-life of 0.5 s. Traditionally, peroxynitrite is detected by examining its downstream product with electrochemical sensors, which often suffer from tedious procedures, inaccuracy, and invisibleness [44]. 

## 4. Fluorescence Imaging-Based Peroxynitrite Detection

Fluorescence imaging is a technique developed in recent years which has been widely applied in the biomedical field. Fluorescent probes generally consist of three moieties: fluorophores, intermediate ligands, and targeting molecules [45]. Fluorescence imaging exhibits advantages such as deep tissue penetration, excellent temporal and spatial resolution, high sensitivity and selectivity, and the ability to achieve real-time visualization [46,47]. Leveraging the merits of fluorescence imaging, fluorescence probes for peroxynitrite have been developed and reported. Herein, we summarize and discuss the currently reported peroxynitrite fluorescence probes in terms of their synthesis, recognition mechanisms, and bioapplications.

### 4.1. Boron-Based Peroxynitrite Probes

Boron and its derivates, including borates, arylborate, and borate ester, can be specifically bound by peroxynitrite, thus serving as a specific recognition moiety of peroxynitrite probes [48,49]. Due to its strong nucleophilicity, boron can be oxidized and hydrolyzed by peroxynitrite to produce phenol derivatives and aromatic nitration, which triggers the generation of a fluorescent signal for peroxynitrite detection. Boronic ester as a response moiety to peroxynitrite had a shorter response time and higher selectivity than other moieties, which could be ascribed to the lower redox potential of borate ester.

In 2020, Dong et al. synthesized a borate-based peroxynitrite fluorescent probe, FAM [50]. In FAM, arylboronic acid esters served as peroxynitrite-recognizing moieties, and anthracycline served as the fluorophore. The lipophilic cation of anthracycline facilitated FAM’s targeting of mitochondria. FAM achieved sensitive and rapid detection of mitochondrial peroxynitrite induced by 3-morpholino-sydnonimine (SIN-1), a donor of peroxynitrite, in RAW 264.7 cells. In 2021, Wang et al. synthesized a benzyl borate-based peroxynitrite fluorescence probe, Probe-L [51]. In Probe-L, coumarin matrix served as the fluorophore. In the presence of peroxynitrite, intramolecular charge transfer (ICT) between the benzenesulfonic acid group and coumarin occurred, and fluorescence was generated. Probe-L distinguished elevated endogenous peroxynitrite in HepG2 liver cancer cells, with neglectable cytotoxicity and high selectivity. In 2021, Dong et al. synthesized an Aggregation-Induced Emission (AIE) fluorescent peroxynitrite probe, ADB [52]. ADB could rapidly and sensitively detect peroxynitrite in RAW 264.7 cells, induced by SIN-1. In the same year, this team designed an arylboronate-based dual functional fluorescent probe, PV [53], which simultaneously detected elevated mitochondrial viscosity and peroxynitrite induced by lipopolysaccharides (LPS) and starvation through twisted intramolecular charge transfer (TICT) in RAW 264.7 cells. Elevated viscosity restricted the rotation of PV and triggered the generation of strong red fluorescence, while peroxynitrite oxidized the arylboronate of PV and induced yellow fluorescence via ICT. PV enabled the detection of zebrafish viscosity induced with nystatin, indicating its potential for in vivo application. 

In 2023, He et al. synthesized a phenylboronate-based peroxynitrite fluorescent probe, TCM-2, with TCF (2-(3-cyano-4,5,5-trimethylfuran-2(5H)-ylidene) malononitrile) as the fluorophore [54]. TCM-2 conjugated with human serum albumin (HSA) via supramolecular host–guest assembly, which enabled more sensitive detection of low concentrations of peroxynitrite in HeLa and RAW 264.7 cells. In 2023, Zhang et al. synthesized a benzeneboronic acid pinacol ester-based peroxynitrite fluorescent probe, BDP-ENE-S-Py^+^, with BODIPY dye serving as the fluorophore [55]. The probe sensitively and specifically detected peroxynitrite induced by SIN-1 in HepG2 cells as well in a mouse model of peritonitis induced by LPS. In 2022, Chen et al. synthesized a two-photon fluorescent peroxynitrite probe, HDBT-ONOO^−^ [56]. In HDBT-ONOO^−^, borate served as the response moiety of peroxynitrite and triphenylphosphine served as the mitochondria-targeting moiety. Once peroxynitrite converted the borate to phenol, red fluorescence was generated. HDBT-ONOO^−^ achieved specific detection of mitochondrial peroxynitrite induced by endogenous and exogenous stimuli in cells and zebrafish. Another group also synthesized a near-infrared peroxynitrite fluorescent probe, P-1, with mitochondria-targeting capacity [57]. Arylboronate of P-1 served as the peroxynitrite-responsive moiety, and quinoline cations served as mitochondria-targeting moieties. P-1 could sensitively detect mitochondrial peroxynitrite induced by SIN-1 in RAW 264.7 cells and zebrafish. 

In 2023, Liang et al. designed and constructed a borate-based chemiluminescence (CL) probe, B-PD, for imaging peroxynitrite [58]. In B-PD, 2-hydroxy-4, 4, 5, 5-tetramethyl-1, 3, 2-dioboropentane served as a specific substrate of peroxynitrite. Acrylonitrile-substituted Schapp’s adamantylidence dioxane (SAD) was designed to enhance the CL quantum yield and prolong the luminous time of the probe. B-PD displayed higher specificity toward peroxynitrite with a longer luminescence lifetime in an inflammatory model induced by LPS and IFN-γ administration in RAW 264.7 cells and mice. In 2023, Li et al. designed a borate ester-based fluorescent probe, NOSTracker, with Si-rhodamine fluorophore (F1) as the fluorophore [59]. NOSTracker itself did not generate fluorescence signals, but in the presence of peroxynitrite, it triggered the conversion of actone into an opening zwitterionic form and induced fluorescence generation. NOSTracker achieved the sensitive and rapid detection of peroxynitrite in U251 cells and in situ glioma of mice. Furthermore, NOSTracker could pass through the blood–brain barrier (BBB), showing high tissue penetration ability. In 2020, James et al. synthesized three kinds of fluorescent probes, CM, CL, and CE, that could detect peroxynitrite of mitochondria, lysosomes, and endoplasmic reticulum, respectively [60]. Those probes were synthesized with coumarin as the fluorophore and phenylboronic acid as a responsive unit, coupled with different organelle-targeting moieties. The fluorescence emission wavelengths of the three probes were 400 nm, 395 nm, and 405 nm, with detection limits of 0.28 μM, 0.26 μM, and 0.36 μM, respectively. The probes detected endogenous and exogenous peroxynitrite in mitochondria, lysosomes, and endoplasmic reticulum of RAW 264.7 cells induced by SIN-1. 

A ratiometric fluorescence probe is an alternative imaging tool that shows two emission signal alterations in response to the target, which could weaken the interference of background signals and other disruptors [61,62]. Hence, a ratiometric probe for peroxynitrite was developed. In 2022, Kang et al. synthesized a near-infrared ratiometric fluorescent probe, JQ-2, for peroxynitrite [63]. In JQ-2, sodium boronate served as the peroxynitrite recognition moiety, and dicyanoisophorone derivatives as the fluorophore. In response to peroxynitrite, the ratio of fluorescence signals in F657 and F569 increased and reached a plateau in around 100 s. The probe specifically detected peroxynitrite in HeLa cervical cancer cells, RAW 264.7 cells, and zebrafish induced by LPS and INF-γ.

### 4.2. C=C- or C=N-Based Peroxynitrite Probes

Conjugated C=C or C=N double bonds can be oxidized and cleaved by peroxynitrite, which leads to the emission of fluorescent signals. In 2021, Kim et al. synthesized a C=C double bond peroxynitrite fluorescent probe, NRF [64]. Peroxynitrite selectively oxidized and cleaved the C=C double bond of hemicyanine and formed indole aldehyde and another product, which possessed a smaller π-conjugation molecular system and therefore emitted fluorescence at 462 nm, accompanied by a decrease in fluorescence at 700 nm. NRF specifically and sensitively detected peroxynitrite in HepG2 cells induced by LPS, IFN-γ, and PMA and in mice induced by SIN-1. Notably, the probe was encapsulated with nanoliposomes, which greatly improved its biocompatibility.

In 2022, Zhang et al. synthesized a ratiometric fluorescence probe for peroxynitrite, MXMP [65]. In MXMP, the C=C double bond between xanthine and pyridine could be oxidized by peroxynitrite, and a fluorescence signal was then released. MXMP sensitively detected peroxynitrite in HeLa cells induced by SIN-1, but its in vivo application was not validated. In 2022, Zhang et al. designed a peroxynitrite fluorescent probe, DMX, with C=N bonds as the peroxynitrite recognition site and xanthene as the fluorophore [66]. The C=N double bond between yellow anthracene and diaminomaleionitrile was oxidized and broken by peroxynitrite, which triggered fluorescence generation. DMX exhibited high sensitivity and selectivity toward peroxynitrite. DMX served as a sensor for monitoring exogenous/endogenous peroxynitrite induced by SIN-1 in HeLa cells and zebrafish. In the same year, Yu et al. synthesized a red fluorescent probe, MCSA, which could specifically detect peroxynitrite in endoplasmic reticulum and mitochondria [67]. In MCSA, indole iodide served as a mitochondria-targeting moiety, and N-(2-aminoethyl)-4-methylbenzenesulfonamide served as an endoplasmic reticulum-targeting moiety. Peroxynitrite oxidized and broke C=C between dihydronaphthalene and indolium iodide, leading to the generation of red fluorescence. MSCA sensitively, selectively, and rapidly detected subcellular peroxynitrite of HeLa, RAW 264.7, and HepG2 cells as well as zebrafish induced by SIN-1, LPS, and IFN-γ, with neglectable cytotoxicity. In 2023, Hu et al. synthesized a ratiometric fluorescence probe for mitochondrial peroxynitrite, COUS [68]. In COUS, the C=C double bond between coumarin aldehyde and 3-ethyl-1,1,2-trimethyl-1H-benzoindole iodide could be recognized and oxidized by peroxynitrite. After reacting with peroxynitrite, coumarin derivatives were released, followed by ratiometric fluorescence alteration. COUS accurately detected peroxynitrite in mitochondria of HepG2 cells, with a large Stokes shift (239 nm) and low detection limit (41.88 nM).

### 4.3. Phosphine-Based Peroxynitrite Probes

In 2018, Zhang et al. synthesized a diphenylphosphonic acid-based peroxynitrite fluorescent probe, CPC [69]. In CPC, quaternized pyridine could target mitochondria due to its positive charge. CPC achieved sensitive and rapid detection of mitochondrial peroxynitrite of HepG2 cells induced by SIN-1, with neglectable cytotoxicity. In 2020, Liu et al. synthesized a near-infrared (NIR) fluorescent peroxynitrite probe, AN-DP [70]. In AN-DP, a phosphine ester moiety served as the recognition moiety of peroxynitrite, with isophorone as the fluorophore. AN-DP could sensitively and rapidly detect peroxynitrite in HepG2 cells induced by SIN-1. In 2020, Ma et al. synthesized a NIR peroxynitrite fluorescent probe, NR-ONOO [71]. Diphenylphosphine in NR-ONOO served as the sensitive and selective recognition moiety of peroxynitrite, and dihydroisophorone served as the fluorophore. In the presence of peroxynitrite, the fluorescence of NR-ONOO was released due to the triggered ICT in dicyanoisophorone. NR-ONOO exhibited high selectivity toward peroxynitrite induced by SIN-1 in HeLa cells. In 2021, Churchill et al. synthesized a diphenyl phosphite-based peroxynitrite fluorescent probe, BICBzDP [72]. BICBzDP was positively charged due to the presence of nitrogen atoms, so it could target mitochondria. The sulfonate group increased the water solubility of BICBzDP. The diphenyl phosphinate moiety served as a peroxynitrite-recognizing moiety. In the presence of peroxynitrite, a donor-p-acceptor (D-π-A) motif was formed and intensive fluorescence was generated. BICBzDP proved to be photostable and could detect peroxynitrite in RAW 264.7 cells induced by LPS and IFN-γ and in zebrafish induced by LPS and Phorbol Myristate Acetate (PMA). In 2021, Shen et al. synthesized a morpholine hydrazone-based peroxynitrite fluorescent probe, BMP [73]. In BMP, benzothiazole served as the fluorophore and morphine served as the lysosome-targeting group. BMP efficiently and specifically detected lysosome peroxynitrite in HeLa cells induced by SIN. In 2022, Cao et al. synthesized a diphenyl phosphonate-based peroxynitrite fluorescence probe, BS1 [74]. BS1 sensitively detected peroxynitrite in HepG2 cells induced by SIN-1. In 2023, Fan et al. synthesized a phosphine ester-based NIR peroxynitrite fluorescent probe, XPC [75]. XPC had a large Stokes shift and showed high sensitivity toward peroxynitrite, with a detection limit of 13 nM. XPC successfully detected endogenous and exogenous peroxynitrite in HeLa cells induced by LPS and PMA.

### 4.4. 1,8-Naphthimide Derivative-Based Peroxynitrite Probes

1,8-naphthalimide and its derivatives show good fluorescence characteristics and optical stability. It was established that peroxynitrite could recognize and react with N-methyl-p-hydroxyaniline so as to release fluorescence [68,76]. In 2022, Wang et al. synthesized a 1,8-naphthalimide-based ratiometric peroxynitrite fluorescence probe, NC-NP_530/460_ [77]. NC-NP_530/460_ enabled sensitive and rapid detection of peroxynitrite in HepG2 and HeLa cells induced by SIN-1, LPS, and IFN-γ.

### 4.5. Other Moiety-Based Peroxynitrite Probes

Besides the aforementioned probes, there are some other moiety-based peroxynitrite fluorescence probes. In 2020, Chao et al. synthesized a 4-[p-methylaniline]-2,2′-bipyridine (MAB)-based NIR fluorescent peroxynitrite probe, Ir-NIR [78]. The dimethylamino groups of NFPQ endowed it with alkalinity, so it could effectively target lysosomes of cells. Ir-NIR could sensitively, rapidly, and selectively detect lysosomal peroxynitrite of RAW 264.7 cells induced by LPS, IFN-γ, and PMA. In 2022, Yuan et al. synthesized a ruthenium (Ru) complex-fluorescein scaffold peroxynitrite probe, Ru-FL-ONOO [79]. In Ru-FL-ONOO, a Ru complex was conjugated to fluorescein, serving as a dual-emissive moiety. The spirocyclic structure of fluorescein-phenylhydrazine served as the specifically reactive moiety of peroxynitrite. Ru-FL-ONOO could selectively and sensitively detect peroxynitrite in HepG2 cells induced by SIN-1. In 2023, James et al. synthesized a 1-methylindoline-2,3-dione-based peroxynitrite fluorescent probe, DSPE-PEG/HN-I [80]. DSPE-PEG/HN-I could sensitively and rapidly detect exogenous and endogenous peroxynitrite in HepG2 and HeLa cells. In 2023, Xu et al. synthesized a squaraine (SQ)-based peroxynitrite probe, SQDC@BCA [81]. Two SQ units were linked by ethylenediamine and subsequently embedded in bovine serum albumin (BSA) to form SQDC-BCA. In the presence of peroxynitrite, one SQ unit was oxidized and the remaining SQ unit entered the hydrophobic cavity of the BSA; subsequently, fluorescence was emitted. SQDC-BCA enabled the detection of peroxynitrite in HepG2 cells induced by LPS. In 2023, Xu et al. synthesized a dihydroquinoline-based peroxynitrite fluorescent probe, probe 1 [82]. Dihydroquinoline was oxidized by peroxynitrite and generated quinoline derivatives. Excited-state intramolecular proton transfer (ESIPT) of the quinoline derivatives triggered aggregation-induced ratiometric emission (AIRE). Probe 1 had a short response time and ultra-sensitivity toward peroxynitrite, and it could monitor the fluctuations of peroxynitrite in HeLa cells and zebrafish induced by LPS. In 2023, Lin et al. synthesized a flavonol-based fluorescent peroxynitrite probe, FLASN [83]. When peroxynitrite was present, ICT of flavonol was restored and generated strong fluorescence. FLASN could not only detect endogenous/exogenous peroxynitrite but also reduce the level of peroxynitrite. It held potential for the diagnosis and treatment of peroxynitrite-related diseases.

### 4.6. Nanoparticle-Based Peroxynitrite Detection 

Besides small-molecule-based peroxynitrite fluorescence probes, in recent years, with the development of nanotechnology, graphene quantum dot (GQD) and carbon dot (CDs)-based probes have also been fabricated and displayed advantages such as high fluorescence intensity, good photostability, light bleaching resistance, and good biocompatibility [84,85]. In 2021, Zhao et al. constructed a ratiometric fluorescent peroxynitrite probe, GQD-Cy5.5, by covalently coupling GQDs with the mitochondria-targeting dye Cy5.5 [86]. The fluorescence emission spectrum of graphene overlapped well with the UV-Vis absorption spectrum of Cy5.5, making them suitable as donors and acceptors of fluorescence resonance energy transfer (FRET). GQD-Cy5.5 was able to detect exogenous and endogenous mitochondrial peroxynitrite in RAW 264.7 cells induced by LPS and IFN-γ. In 2022, Lin et al. synthesized a CDs and phycocyanin (PC)-based ratiometric fluorescent nanoprobe for peroxynitrite, PC-CDs [87]. In the presence of peroxynitrite, the CDs generated fluorescence with a peak at 450 nm, while that from PC had a peak at 645 nm. PC-CDs could selectively detect exogenous peroxynitrite of MC3T3-E1 cells. In 2024, James et al. synthesized another ratiometric fluorescent peroxynitrite probe, CD-N-I [88]. In CD-N-I, CDs served as energy donors and a naphthalimide fluorophore served as an energy acceptor to obtain an effective FRET platform. Peroxynitrite triggered the activation of the isatin receptor and activated the FRET process. CD-N-I enabled sensitive and selective detection of peroxynitrite in HepG2 cells induced by SIN-1.

The details of the aforementioned peroxynitrite probes in terms of their structure, excitation/emission, and working mechanism/bioapplication are summarized in Table 1. 

## 5. Application of Fluorescent Probes in PD Study

**Figure 3:**
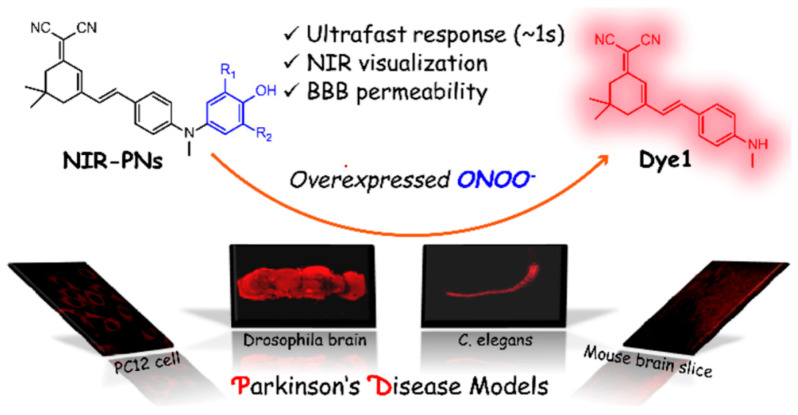
Schematic strategy of developing and applying NIR-PNs for detecting peroxynitrite in PD models (adapted from reference [89] with permission).

Given the aforementioned merits of peroxynitrite fluorescence probes and the pivotal role of peroxynitrite in PD pathogenesis, it was of great value to apply those probes in PD studies. In 2020, we synthesized a dicyanoisophorone-based NIR fluorescence probe for peroxynitrite, NIR-PN1 [89] (Figure 3). NIR-PN1 was constructed with dicyanoisophorone as a donor (D) and p-aminophenol as an acceptor (A). NIR-PN1 had high selectivity and sensitivity and a short response time for peroxynitrite. The fluorescence of the probe was quenched by photoinduced electron transfer (PET). After reaction with peroxynitrite, the p-aminophenol group was cleaved to release benzoquinone, resulting in fluorescence restoration. NIR-PN1 could monitor endogenous peroxynitrite in PC12 cells and SH-SY5Y cells. Noteworthily, NIR-PN1 could image the elevated peroxynitrite levels in various types of PD models, including cells, drosophila, C. elegans, and mice, indicating that the NIR-PN1 probe could be used to monitor peroxynitrite fluxes in vitro and in vivo in the context of PD.

**Figure 4:**
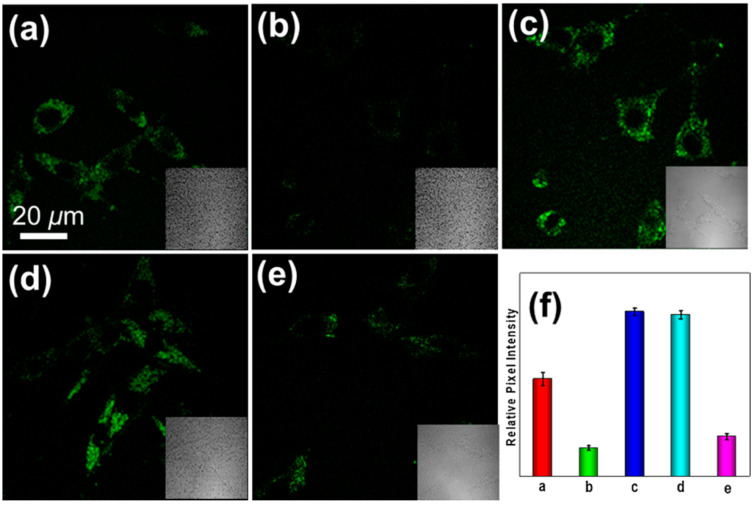
Two-photon fluorescence imaging of peroxynitrite with ER-PN in PC12 cells. (**a**) Sham control cells incubated with probe ER-PN. (**b**) Cells pre-treated with UA and subsequently incubated with ER-PN. (**c**) Cells pre-treated with SIN-1 and then incubated with ER-PN. (**d**) Cells subjected to LPS/IFN-γ for 12 h and subsequently incubated with ER-PN. (**e**) Cells pre-treated with UA, subjected to LPS/IFN-γ, and subsequently incubated with ER-PN (adapted from reference [90] with permission). (**f**) is a collection of subfigure (**a**)–(**e**).

In 2021, we developed a two-photon fluorescence probe, ER-PN, for detecting peroxynitrite in the ER [90]. In ER-PN, 4-amino-2-methoxyphenol (AP) served as the recognizing moiety of peroxynitrite, and toluene sulfonamide worked as the ER-targeting moiety. ER-PN exhibited weak fluorescence due to the presence of PET, and after reacting with peroxynitrite, the AP group cracked and exhibited strong fluorescence. ER-PN displayed high selectivity toward peroxynitrite and enabled bioimaging of the dynamic changes in peroxynitrite in PC12 cells (Figure 4) and zebrafish PD models, with good biocompatibility. It showed great potential in the diagnosis of PD. In 2022, we designed a novel ketoamide-based peroxynitrite fluorescence probe, DFlu [91]. Naphthylamine ketone served as a two-photon fluorophore of DFlu, which avoided autofluorescence interference and minimized photodamage to organisms during imaging. After reacting with peroxynitrite, the amide bond of the ketoamide was broken and a fluorescence signal was triggered. DFlu detected endogenous and endogenous peroxynitrite in SH-SY5Y and a zebrafish PD model induced by neurotoxins (Rotenone and MPP^+^). To improve the detection sensitivity and accuracy, Zhang et al. synthesized an NIR ratiometric fluorescent probe, K-ONOO [92]. In K-ONOO, borate ester served as the recognition moiety of peroxynitrite, and a dicyanoisophorone derivative with the donor (D)-π-acceptor (A) structure served as the fluorophore. Peroxynitrite triggered cleavage of the borate portion of the probe, and the probe exhibited a strong redshift emission due to ICT. K-ONOO could sensitively and rapidly detect peroxynitrite in cellular and zebrafish PD models. In 2024, Tang et al. synthesized a peroxynitrite fluorescent probe, DHX-SP [93]. In DHX-SP, phenylborate and pyridinium were employed as the peroxynitrite recognition units, atrifluoromethanesulfonate served as the chemoselective O_2_·^−^-cleavable moiety, and rigid dihydroxanthene served as the fluorophore. DHX-SP exhibited high selectivity and sensitivity toward both O2·^−^ and peroxynitrite. In a 6-OHDA and ferroptosis activator (Erastin)-induced PC12 cellular PD model, DHX-SP enabled effective visualization of both O_2_·^−^ and peroxynitrite. 

Given that PD shares a common pathogenesis mechanism with Alzheimer’s disease (AD), namely nitrosative stress, peroxynitrite fluorescence probes were also applied in AD research. In 2021, Tang et al. synthesized a two-photon probe, BTNPO. Here, oxindole specifically reacted with peroxynitrite to generate an aniline derivative, which induced a bathochromic shift due to the strengthened ICT and generated fluorescence. BTNPO could simultaneously detect elevated Aβ protein and peroxynitrite levels in cellular and mouse AD models [94]. The following year, this group reported anther peroxynitrite fluorescence probe, NATP. Upon peroxynitrite treatment, NATP experienced oxindole decyclization and formed an aniline derivative, which was accompanied by fluorescence activation owing to the enhanced ICT effect. NATP enabled the detection of peroxynitrite in an Aβ-induced cellular AD model as well as in AD mouse brains due to its capacity to penetrate the BBB [95]. In 2022, Kim et al. designed a peroxynitrite NIR fluorescence probe, Rd-DPA3 [96]. Rd-DPA3 showed high sensitivity toward peroxynitrite and enabled its detection in PC12 cells and a mouse AD model. In 2023, Wu et al. constructed a long wavelength peroxynitrite probe, DCO-POT [97]. DCO-POT had a large Stokes shift, which helped to avoid the interference of spontaneous fluorescence. Peroxynitrite oxidized the C=C bond of DCO-POT, and the fluorophore, DCO-CHO, generated fluorescence due to the ICT effect. DCO-POT was successfully applied to image peroxynitrite in living cells and a zebrafish AD model. 

## 6. Conclusions and Prospectives

It is well recognized that nitrosative stress is a crucial causative factor of PD and other degenerative diseases, and peroxynitrite is the prime initiator of nitrosative stress. It is therefore of great significance to reveal the mechanism of peroxynitrite underlying PD pathogenesis by visually monitoring its dynamics. In the present review, we firstly introduced the background on PD and peroxynitrite. Then, we depicted the possible pathways though which peroxynitrite is involved in PD pathogenesis. After that, we summarized the fluorescence probes for peroxynitrite reported to date and deliberately addressed their working mechanisms for peroxynitrite detection as well as bioimaging applications. Finally, we highlighted the development and application of fluorescence probes in the context of PD. The present review not only broadens the knowledge about the physiopathological role of peroxynitrite but also provides valuable insights for exploring feasible tools for peroxynitrite detection.

Notably, there are still existing gaps to be filled. Firstly, the precise molecular mechanism by which peroxynitrite leads to PD remains to be elucidated with the help of multi-omics. Secondly, probes with higher specificity, sensitivity, and biocompatibility are necessary. Due to the low concentration of peroxynitrite in complex biological environments and its short half-life, there is an urgent need to design probes with better sensitivity and selectivity. Moreover, the reaction mechanisms of the currently available probes are primarily based on oxidative reaction with boronic acids and their ester derivatives, or oxidation-induced cleavage of ethylenic bonds, which cannot avoid the potential interference of other active species such as H_2_O_2_. Thirdly, factors such as good solubility, cell permeability, and low interference of the biological environment should be considered in the design of the probe. Lastly, the clinical application of the reported peroxynitrite probes needs to be verified. With the rapid development of biology, chemistry, and technology, these issues will hopefully be settled in the near future. 

## Figures and Tables

**Table 1:** Probes used for the detection of peroxynitrite.

Serial Number	Probe Structure	Excitation/Emission (nm)	Working Mechanism/Application	Reference
1 (FAM)	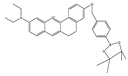	580/625	Boron-based/cells	Dong et al. [50]
2 (L)	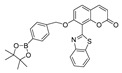	421/500	Boron-based/cells	Zhu et al. [51]
3 (ADB)	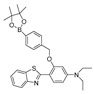	362/440	Boron-based/cells	Dong et al. [52]
4 (PV)	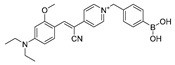	447/507	Boron-based/cells and mice	Dong et al. [53]
5 (TCM-2)	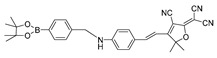	-/560	Boron-based/cells	He et al. [54]
6 (BDP-ENE-S-Py+)	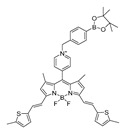	620/640,900	Boron-based/cells, mice	Zhang et al. [55]
7 (HDBT-ONOO^−^)	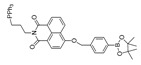	372,450/558	Boron-based/cells, zebrafish	Chen et al. [56]
8 (P-1)	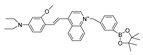	358/570	Boron-based/cells, zebrafish	Shuang et al. [57]
9 (B-PD)	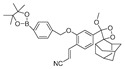	-	Boron-based/cells, zebrafish	Liang et al. [58]
10 (NOSTracker)	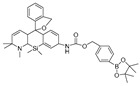	645/696	Boron-based/cells, mice	Li et al. [59]
11 (CM)	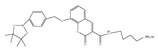	340/400	Boron-based/cells	James et al. [60]
12 (CL)	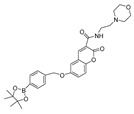	340/395	Boron-based/cells	James et al. [60]
13 (CE)	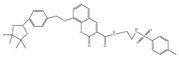	340/405	Boron-based/cells	James et al. [60]
14 (JQ-2)	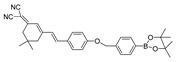	461/569,657	Boron-based/cells, zebrafish	Kang et al. [63]
15 (NRF)	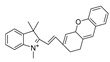	462/700	C=C-based/cells, mice	Kim et al. [64]
16 (MXMP)	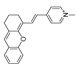	390,520/530,660	C=C-based/cells	Zhang et al. [65]
17 (DMX)	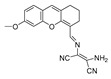	475/510	C=N-based/cells, zebrafish	Zhang et al. [66]
18 (MCSA)	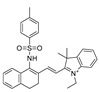	540/635	C=N-based/cells, zebrafish.	Yu et al. [67]
19 (COUS)	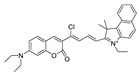	363,586/484,723	C=C-based/cells	Hu et al. [68]
20 (CPC)	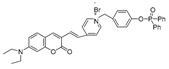	503/538,643	Phosphine-based/cells	Zhang et al. [69]
21 (AN-DP)	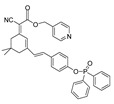	454/670	Phosphine-based/cells.	Liu et al. [70]
22 (NR-ONOO)	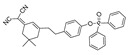	400/560	Phosphine-based/cells	Ma et al. [71]
23 (BICBzDP)	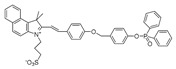	549/576	Phosphine-based/cells, zebrafish	Churchill et al. [72]
24 (BMP)	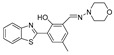	427/515	Phosphine-based/cells	Shen et al. [73]
25 (BS1)	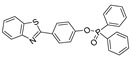	365/430	Phosphine-based/cells	Cao et al. [74]
26 (XPC)	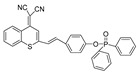	550/750	Phosphine-based/cells	Fan et al. [75]
27 (NC-NP530/460)	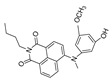	405/460,530	1,8-naphthimide derivative-based/cells	Wang et al. [77]
28 (Ir-NIR)	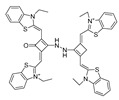	405/702	MAB-based/cells	Chao et al. [78]
29 (Ru-FL-ONOO)	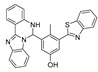	490/635	Fluorescein-phenylhydrazine-based/cells, zebrafish	Yuan et al. [79]
30 (DSPE-PEG/HN-I)	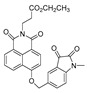	448/563	1-methylindoline-2,3-dione/cells	James et al. [80]
31 (SQDC)	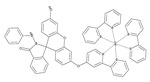	630/685	Squaraine-based/cells	Xu et al. [81]
32 (probe 1)	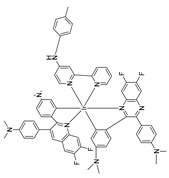	365/485,545	Dihydroquinoline-based/cells, mice	Xu et al. [82]
33 (FLASN)	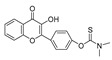	405/525	Flavonol-based/cells, zebrafish, mice	Lin et al. [83]
34 (CD-N-I)	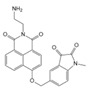	245,350/400	CDs-based/cells	James et al. [88]
35 (NIR-PN1)	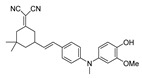	511/670	Dicyanoisophorone-based/cells, drosophila, *C. elegans*, mice	Liu et al. [89]
36 (ER-PN)	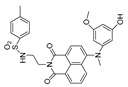	450/540	AP-based/cells, *C. elegans*	Li et al. [90]
37 (DFlu)	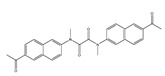	313/490	Naphthylamine ketone-based/cells, zebrafish	Li et al. [91]
38 (K-ONOO)	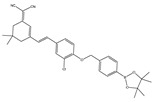	405,525/570,678	Borate ester-based/cells, zebrafish	Zhang et al. [92]
39 (DHX-SP)	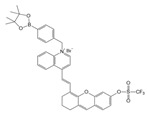	470/560	Phenylborate and pyridinium-based/cells	Tang et al. [93]
40 (NATP)	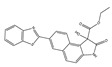	390,450/565	Oxindole/cells, mice	Tang et al. [94]
41 (BTNPO)	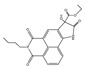	380/506	Oxindole/cells, mice	Tang et al. [95]
42 (Rd-DPA3)	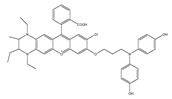	564/698	Azanediyldiphenol-based/cells, mice	Kim et al. [96]
43 (DCO-POT)	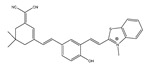	500/670	C=C/cells, zebrafish	Wu et al. [97]
